# The effect of self-directed online metacognitive listening practice on Chinese EFL learners' listening ability, metacognition, and self-efficacy

**DOI:** 10.3389/fpsyg.2023.1285059

**Published:** 2023-11-09

**Authors:** Tao Pei, Jitpanat Suwanthep, Huashan Lu

**Affiliations:** ^1^School of Foreign Languages, Shaoguan University, Shaoguan, China; ^2^School of Foreign Languages, Suranaree University of Technology, Nakhon Ratchasima, Thailand; ^3^School of Foreign Languages, Qingdao Agricultural University, Qingdao, China

**Keywords:** online learning, metacognitive instruction, L2 listening, metacognitive awareness, self-efficacy

## Abstract

Research into metacognitive listening instruction under the Metacognitive Pedagogical Cycle (MPC) has been growing in recent decades, but its effects on L2 listening comprehension, metacognitive awareness, and self-efficacy remain inconclusive. In this mixed-method study, we developed a self-directed online listening practice based on the MPC and investigated its effects on 89 Chinese intermediate EFL learners over 14 weeks. Learners were assigned to either an experimental group, which used the online metacognitive listening practice, or a control group, which used the traditional listening practice without stressing metacognitive awareness. Multiple data sources (listening tests, questionnaires, reflective notes, and interviews) were used to assess learners' listening comprehension, metacognitive awareness, and listening self-efficacy. Results showed that online metacognitive listening practice significantly improved the learners' listening comprehension, but there was little evidence that it increased metacognitive awareness or listening self-efficacy. This study suggests that deploying online listening practice under MPC is a more effective way to improve L2 learners' listening comprehension than traditional listening practice. However, the task-setting of MPC and the task-dependence of self-efficacy may constrain the development of some factors of metacognitive awareness and self-efficacy.

## 1. Introduction

Listening is the first language skill that children acquire, preceding speaking, reading, and writing. Most children can develop listening ability naturally without formal instruction (Siegel, 2015). However, L2 (second language) listening is a complex and daunting task that imposes on language learners high cognitive demands (Satori, 2022; Zhang and Shen, 2023). L2 learners need to use higher-order cognitive abilities, such as metacognitive strategies, to bridge listening comprehension gaps and achieve listening success (Vandergrift, 2003; Norris et al., 2017; Goh and Vandergrift, 2021). To help develop listening skills, Vandergrift (2004, 2007) proposed Metacognitive Pedagogical Cycle (MPC), the metacognitive instruction approach that helps learners regulate their listening processes and develop strategies for improving listening comprehension. Recent studies (e.g., Graham and Macaro, 2008; Taguchi, 2017; Ahmadi Safa and Motaghi, 2021; Razavi et al., 2023) have examined the effects of the MPC on L2 listening and related cognitive factors (e.g., metacognitive awareness and self-efficacy) but the results have been inconclusive. Therefore, further investigation into the effect of the MPC is warranted. In addition, technological advancement has provided learners with new opportunities for L2 listening. Learners now have access to a wider range of listening resources and more chances to listen in real contexts. As a result, the focus of L2 listening is shifting from class-based teaching to self-directed learning (Vandergrift, 2007). Therefore, it is necessary to incorporate metacognitive activities into a technology-enhanced listening environment (Goh and Vandergrift, 2021; Bozorgian and Shamsi, 2023). However, rare research has examined the integration of MPC with technology and tested its effect on L2 listening ability.

In this study, we developed a self-directed online listening practice package based on the MPC and, using a mixed-method study, tested its effectiveness on Chinese university learners' listening comprehension, metacognitive awareness, and self-efficacy. The online listening practice package was designed to include necessary steps in the MPC, such as prediction, monitoring, and reflection. The package was then compared with a traditional online listening practice package, which simply involved listening and answering questions or summarizing the content. The standardized listening test was used to measure L2 listening ability, and the metacognitive awareness listening questionnaire (MALQ) (Vandergrift et al., 2006) and listening self-efficacy questionnaires (LSQ) (Graham and Macaro, 2008) were used to gauge metacognitive awareness and self-efficacy. Qualitative data from reflective notes and post-interviews were also collected.

## 2. Related work

### 2.1. Research on metacognitive instruction

Metacognition is how people's cognitive processes are monitored, regulated, and orchestrated (Flavell, 1979). In recent decades, there has been a surge in metacognitive instruction studies in the L2 listening field (e.g., Vandergrift and Tafaghodtari, 2010; Cross, 2011; Bozorgian, 2014; Fahim and Fakhri, 2014; Wang, 2016). Many of these are designed based on the MPC (Vandergrift, 2004, 2007), which combines listening tasks with metacognitive activities and takes learners through the metacognitive processes of planning, monitoring, evaluation, and problem-solving in listening. Accordingly, an increasing body of evidence supports the effects of metacognitive instruction on L2 listening comprehension. For example, Vandergrift and Tafaghodtari (2010) investigated the impact of metacognitive instruction grounded in the MPC for 14 weeks. Less-skilled listeners in the metacognitive group significantly outperformed the control group in developing listening comprehension and one factor (Problem-solving) associated with metacognitive awareness. They indicated that learners' regular engagement in metacognitive processes facilitated the formation of implicit knowledge of strategies, thus promoting strategy use. Similarly, Bozorgian (2014) studied metacognitive instruction's effect on Iranian L2 learners' listening comprehension. After 8-week intervention, the learners made a significant improvement in listening comprehension and two factors associated with metacognitive awareness (Planning-evaluation and Problem-solving). Alamdari and Hosnbakhshan (2021) investigated the effects of metacognitive instruction delivered separately in L1 and L2 for Iranian upper-intermediate L2 learners. The results showed that metacognitive instruction with L1 delivery produced the most gains in listening comprehension and metacognitive awareness.

However, inconclusive evidence has also been reported. Wang (2016) examined metacognitive instruction with Chinese university EFL listeners. The study did not support the superiority of metacognitive instruction as compared to traditional instruction in enhancing listening ability. Nevertheless, the journal data showed that learners made certain improvement in metacognitive knowledge. In the present study and in the study of Wang (2016), “traditional” listening instruction is characterized as the comprehension approach (Field, 2008), in which learners listen several times (usually three times) and, with or without guidance from teachers, check their performance with comprehension questions or other tasks (e.g., the summary). “Traditional” listening instruction focuses on the listening result rather than the process and does not usually aim to enhance learners' metacognitive awareness. In addition, Taguchi (2017) failed to find a treatment effect for metacognitive instruction in developing Japanese EFL learners' listening comprehension. The author further pointed out that listeners without adequate listening practice may not benefit from metacognitive instruction, due to the lack of necessary bottom-up and top-down skills. As a result of these conflicting results, more research is required to assess metacognitive instruction in different settings and with more variables, such as self-efficacy.

### 2.2. Self-efficacy and L2 listening

Self-efficacy refers to one's own judgment of one's ability to complete a specific task and achieve the desired performance (Bandura, 1977), and “the control over the events that affect their lives” (Bandura, 1989, p. 1175). Self-efficacy beliefs can influence human motivations, achievements, and psychological wellbeing (Bandura, 1992). Given that the processes involved in L2 listening are difficult for learners to control, listening in L2 often results in a reduction in positive feelings of self-efficacy in learners (Graham, 2011). Thus, improving self-efficacy may be important to developing listening ability. Furthermore, previous studies (e.g., Chen, 2007; Rahimi and Abedi, 2014) demonstrated the strong links between self-efficacy, metacognition, and listening ability. Metacognition can regulate the relationships between self-efficacy and listening comprehension (Siegel, 2014). Self-efficacy and metacognition conceptually overlap since one metacognitive awareness factor, namely, Person Knowledge comprises “self-concept and self-efficacy” (Goh and Vandergrift, 2021, p. 92). Therefore, it is reasonable to expect metacognitive instruction to improve listening self-efficacy (Graham, 2011).

However, the evidence concerning the contribution of metacognitive instruction to self-efficacy development needs to be clarified. Graham and Macaro (2008) investigated the impact of strategy training with writing feedback on the listening ability and self-efficacy of L2 French learners. This study can be viewed as a metacognitive intervention (Cross, 2015) as it involved diaries and feedback. The results showed that the learners who received the metacognitive intervention reported stronger self-efficacy beliefs than those who did not. The authors indicated that writing feedback can help learners reflect on their metacognitive knowledge and strategy use. However, Taguchi (2017) found that Japanese EFL learners improved self-efficacy beliefs under metacognitive and traditional instruction, and no significant between-group differences were observed. The author attributed the improvement in self-efficacy to the increased listening practice that both groups engaged in, as more listening practice can contribute to more successful listening experiences. Similarly, Milliner and Dimoski (2021) examined the impact of the metacognitive intervention on Japanese EFL learners' listening self-efficacy. The results showed no significant between-group differences in the listening self-efficacy scores between the metacognitive and traditional groups after training. Despite this, within-group differences indicated that the treatment did help the experimental group improve self-efficacy, but its advantage was “slightly” displayed (p. 1). Hence, given the lack of strong evidence concerning the benefits of metacognitive instruction in enhancing self-efficacy, more research is required.

### 2.3. Online L2 listening research

Online learning provided new opportunities for L2 listening. For instance, learners can control their listening pace by regulating speech rates and delivery ways (Robin, 2007). They also have the opportunity to listen multiple times and access a large collection of listening resources anytime and anywhere with a stable connection. Moreover, online learning can compensate for teachers' knowledge gaps concerning how to teach listening skills (Chen and Zhang, 2010).

However, research on the effects of online learning on L2 listening comprehension has produced mixed results. Certain studies (e.g., Smidt and Hegelheimer, 2004; Absalom and Rizzi, 2008) found the advantage of online listening tasks, while others (Chen and Zhang, 2010) did not. Absalom and Rizzi (2008) compared online listening tasks to text-based listening tasks with L2 Italian learners. The results showed that online listening tasks can contribute to increased vocabulary and information retention than text-based tasks. However, in the study of Chen and Zhang (2010), instruction with online learning systems did not surpass traditional listening instruction in improving Chinese EFL learners' listening comprehension. Thus far, few studies have delved into metacognitive intervention in an online setting, especially drawing on the Metacognitive Pedagogical Cycle. Barbosa-Hernández (2012) investigated the effects of metacognitive strategy instruction with online listening activities on Colombian EFL learners' listening ability. Using questionnaires, journals, and interviews, the study showed that teaching metacognitive strategies online can help students listen more carefully. For students who lacked time to participate in face-to-face courses, such instruction was especially useful.

In summary, given the inconclusive results regarding the effects of metacognitive instruction and online listening, together with the paucity of research combining both, the study attempted to fill this void by investigating the effects of online metacognitive listening practice on L2 learners' listening comprehension, metacognitive awareness, and self-efficacy. Specifically, the study aims to answer the following research questions:

- To what extent does the online metacognitive listening practice improve Chinese EFL learners' listening comprehension?
- To what extent does the online metacognitive listening practice improve Chinese EFL learners' metacognitive awareness?
- To what extent does the online metacognitive listening practice improve Chinese EFL learners' listening self-efficacy?

## 3. Method

### 3.1. Research design

This study used a pre-test–post-test control group quasi-experiment design, with a mixed research method. Quantitative and qualitative data were gathered through listening tests, questionnaires, reflective notes, and interviews. The independent variable was the metacognitive approach of online listening practice in the experimental group (vs. the traditional approach in the control group). The dependent variables were the listening comprehension ability, metacognitive awareness, and listening self-efficacy. Since both the experimental and control groups took classroom-based listening courses taught by the same teacher, this classroom-based instruction variable was controlled for.

### 3.2. Participants

The study participants were 89 Chinese first-year university EFL learners (8 males and 81 females) from two intact classes, with an average age of 20, ranging from 19 to 22. They were at the intermediate level (CEFR B2 level) at the time of research. From the original recruitment (*N* = 100) from the two classes, 11 students dropped out of the final data analysis as they failed to finish all questionnaires or tests. The two classes were randomly divided into experimental and control groups. Before the study, participants signed informed consent forms and were informed of participating in a study to improve their listening comprehension and the freedom to withdraw.

Moreover, we gained the instructor's permission to conduct a study in her classes, but we did not reveal the specific treatment or research questions to her. She agreed to use the online listening activities as required assignments for students (rather than as optional extra credits) to ensure the student participants engaged in the activities. To control for the in-class interventions, the researchers observed the instructor's lessons several times before the study and found that she mainly used a comprehension approach (Field, 2008) in listening instruction. The typical lesson she taught consisted of the activities of listening, answering questions, and checking, without an attempt to raise metacognitive awareness.

### 3.3. The self-directed online listening practice package

The self-directed online listening practice includes 28 sets of listening practice exercises (for 14 weeks), each of which was designed based on the Metacognitive Pedagogical Cycle (MPC) (Vandergrift, 2004, 2007), as shown in Table 1. Since holding synchronous discussion sessions is difficult in the online listening context, in the current online listening practice, we removed the discussion part from the MPC but included an extra biweekly reflection stage. Given both discussion and reflection can be used to evoke learners' metacognitive knowledge (Goh, 2008), this replacement was deemed appropriate. Furthermore, we added a sentential dictation practice exercise based on the listening transcript in the fourth stage of the listening practice to integrate more bottom-up listening practice in the MPC, as suggested in the study of Vandergrift and Tafaghodtari (2010).

**Table 1:** The stages of online metacognitive listening practice.

**Stages**	**Metacognitive processes**
**Pre-listening—Planning stage**	
Learners read the topic and related words. Then, they answered some questions to plan their listening goals (strategy use and potential listening problems), and made predictions related to information and possible words.	Planning
**First listening—First verification stage**	
Learners verified their initial hypotheses, made corrections as required, and noted additional information as they understood it (they evaluated the effectiveness of strategies and planned new strategies for the second listening).	Monitoring and evaluation
**Second listening—Second verification stage**	
Learners listened again and supplemented the information missed in the first listening (and then evaluated the effectiveness of listening strategies for the second listening and the degrees of comprehension). Then, they answered some listening comprehension questions and summarized the main contents of the listening text. After completion, they checked their answers.	Monitoring, evaluation, and problem-solving
**Third listening—Final verification stage**	
Learners completed the sentential dictation tasks and checked the transcripts. Then, they wrote down difficult words from the listening (and evaluated the difficulty level of the listening materials and their general performance).	Evaluation and problem-solving
**Reflection stage**	
Learners summarized listening problems and useful strategies in the listening process and planned strategies for the next listening.	Evaluation and problem-solving
**Further reflection stage (biweekly)**	
Once every 2 weeks, learners took reflective notes on learning difficulties, perceived changes in their listening ability, strategy use, listening confidence, and any other thoughts.	Evaluation and problem-solving

To increase the diversity of practice exercises, the second set of listening practice exercises each week did not involve the bracketed contents.

The online listening practice was delivered through the online questionnaires embedded in web pages. The multi-page layout of the questionnaire matched the multi-stage structure of metacognitive listening. We published two sets of listening practice exercises on a website every week for learners to complete. The listening content contained 3–5 min videos of news and lectures (without subtitles) at a normal speech rate (around 140 wpm) and the topics were in line with those of the in-class listening textbooks. Learners were required to enter their names once they started listening practice, and the researchers could check their responses in the online admin panel. To increase motivation, we received the instructor's permission to combine the practice attendance with the final credits of the in-class listening course.

### 3.4. Instruments

#### 3.4.1. TEM-4 test

The listening comprehension ability was gauged with the listening sections of two actual TEM-4 (Test for English Majors—Band 4) tests, as the pre-and post-tests. As a nationwide English placement test in China, TEM-4 was familiar to the participating students. Since its debut in 1992, the TEM-4 test has been regularly validated (Jin and Fan, 2011). A Sino-British cooperative validation study conducted between 1993 and 1996 demonstrated that the test was valid and had a Cronbach's alpha of 0.85 (The TEM Test Center, 1997, p. 63). The listening section of the TEM-4 test consisted of the following: (a) three long conversations followed by nine multiple-choice (MC) questions; (b) three monologs followed by nine MC questions; and (c) five short pieces of news followed by 11 MC questions. According to a pre-research survey, the participants had not previously practiced the two sets of TEM-4 tests used (as pre-and post-tests) in the study.

#### 3.4.2. Questionnaires

The study used the Metacognitive Awareness Listening Questionnaire (MALQ) and Listening Self-efficacy Questionnaire (LSQ) to measure the learners' metacognitive awareness and listening self-efficacy. MALQ was developed and validated by Vandergrift et al. (2006). The questionnaire uses a 5-point Likert scale comprising 21 items covering five factors: Planning-evaluation, Directed Attention, Person Knowledge, (no) Mental Translation, and Problem-solving. Previous studies (e.g., Vandergrift and Tafaghodtari, 2010; Rahimi and Katal, 2013; Bozorgian and Alamdari, 2017; Mahdavi and Miri, 2019) showed that the MALQ's reliability was above an acceptable level, with Cronbach α > 0.7.

The Listening Self-efficacy Questionnaire (LSQ), adapted from Graham and Macaro (2008), was used to measure listening self-efficacy. This questionnaire appraised listeners' beliefs in their ability to manage specific listening comprehension skills and reflected the task-specific nature of self-efficacy. The questionnaire uses a 100-point scale with four questions and is highly reliable, as reported in a study by Milliner and Dimoski (2021), with a Cronbach α of 0.89. This questionnaire was given immediately after the listening test to narrow down the delay between self-efficacy beliefs and listening performance.

The original MALQ and LSQ were translated into Chinese by two professional translators using a forward-backward translation technique (Lee et al., 2019). Before the study, the two questionnaires were tested with a cohort of 50 non-participants and exhibited acceptable reliability levels, with Cronbach α values of 0.89 and 0.85, respectively.

#### 3.4.3. Reflective notes and interviews

As part of the metacognitive listening practice, the experimental group took biweekly reflective notes during the listening practice (a total of seven times over 14 weeks). Before the experiment, the learners were given prompts for writing notes. They were encouraged to note down learning difficulties, perceived changes in listening ability, strategy use, listening confidence, and any other thoughts for every 2 weeks of practice. The researchers reviewed the notes and provided feedback to each participant to help connect their performance and strategy use (Graham and Macaro, 2008). These reflective notes were collected with online questionnaires, and feedback was sent to each learner via the QQ instant messenger.

Additionally, around 30% of participants from the experimental group met with the researchers for semi-structured interviews after the training. During interviews, they expressed their thoughts on their improved listening abilities, strategies, confidence, and perceptions of listening practice. The interviews lasted around 5–10 min for each participant.

### 3.5. Procedures

The study lasted one semester, from March 2022 to July 2022, which is around 16 weeks. Week 1 and week 16 were scheduled to administer listening tests, the MALQ, and the LSQ. In the first week, the researchers conducted a single 90-min session to introduce each group to perform the online listening practice package. In week 16, 15 learners were randomly chosen from the experimental group to join the post-interview.

Each week throughout the 14 weeks, the experiment group was assigned two sets of online listening practice exercises on a webpage. The first set followed the arrangement shown in Table 1. To increase the diversity of practice methods, the second set was more concise and included fewer questions than the first set but still engaged learners in the metacognitive processes of planning, monitoring, and evaluation. Learners kept reflective notes every 2 weeks and sent them to the researchers, who returned feedback to each learner within the same week.

The control group was assigned a traditional form of online listening practice package, in which they listened to the same materials three times and then answered comprehension questions or wrote a summary with sentence dictation tasks. In addition, given the increased number of activities in the experimental training, the control group was also required to listen to two extra texts and answer multiple-choice comprehension questions to ensure a comparable level of involvement in listening practice. Both groups could contact the researchers if they encountered any problems during the practice. The researchers occasionally checked the learners' responses to ensure they had completed the practice in a focused manner. The researchers sent personal notifications to ensure the engagement of learners who forgot the practice or who seemed to produce the answers hastily.

### 3.6. Data analysis

The IBM SPSS 24 software was used to analyze data from the listening tests (TEM-4 tests), the MALQ, and LSQ. We conducted ANCOVA tests to measure the group effects between the two groups with the pre-test scores as the covariates. Learners' responses in reflective notes and interviews were coded and analyzed in the Nvivo11 software. To increase coding reliability, two researchers of the study coded 50% of the data separately and reached an inter-coder agreement at 88%, which is an acceptable level. After negotiating with the second researcher, the first researcher coded the rest of the data and formulated the final themes.

## 4. Results

### 4.1. Listening test results

**Figure 1:**
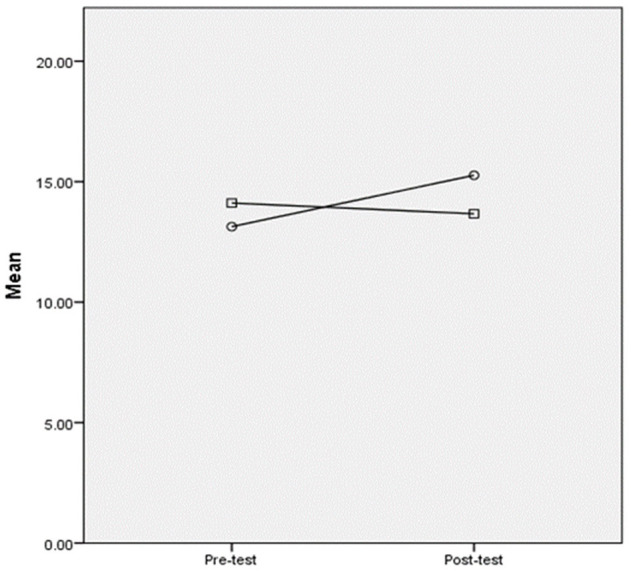
Plot comparing the pre-and post-test listening scores. ° = The experimental group; □ the control group.

Descriptive analysis was conducted for the pre-and post-listening test scores, as shown in Table 2. The pre-and post-test listening scores met the assumption of homogeneity of variance in Levene's test of equality (*p* = 0.15; *p* = 0.73). According to Figure 1, as compared with the post-test, only the experimental group demonstrated an increase in post-listening scores while the control group showed a slight decrease. A similar result has been reported in the literature. For instance, in the study by Milliner and Dimoski (2021), the control group and strategy group also experienced a decrease in listening scores after the training, which implies that enhancing listening abilities through practice is not easy and may require a substantial amount of time. Thereafter, we ran an ANCOVA test to examine the group effect with the pre-test scores as the covariate and the post-test as the dependent variable. According to the results of the ANCOVA test (see Table 3), the results indicated a significant group effect (*p* = 0.004) with a medium effect (η^2^ = 0.09), suggesting that the online metacognitive listening practice can significantly improve the learners' L2 listening comprehension ability.

**Table 2:** Descriptive statistics of the listening scores.

**Tests**	**Experimental group (*****n*** = **45)**		**Control group (*****n*** = **44)**	
	***M***	***SD***	***M***	***SD***
Pre-test	13.13	2.35	14.11	3.42
Post-test	15.27	2.6	13.67	3.07

**Table 3:** ANCOVA results of the group effect on the listening scores.

**Source**	***df***	**Mean square**	***F***	***p***	**η^2^**
Group	1	71.11	8.92	0.004^**^	0.09
Pre-test	1	35.74	4.49	0.03^*^	0.05
Residual	86	7.97			

* p < 0.05.

** p < 0.01.

### 4.2. Questionnaire results

#### 4.2.1. MALQ results

**Figure 2:**
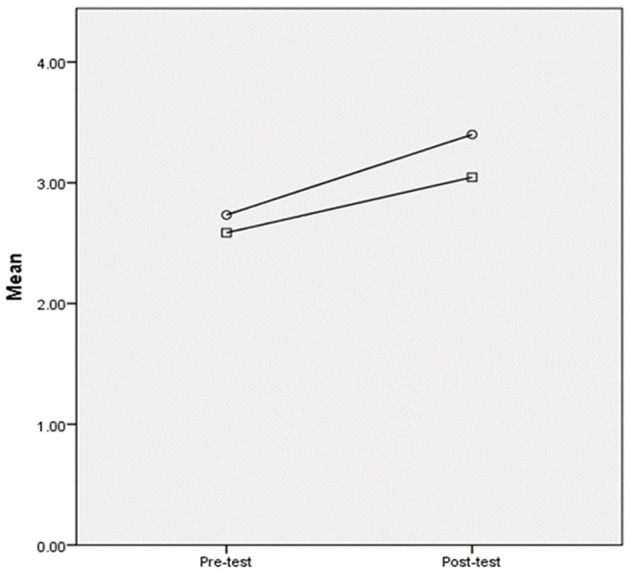
Plot comparing the pre-and post-test scores of the planning-evaluation factor. ° = The experimental group; □ the control group.

Table 4 shows the descriptive analysis of the MALQ results for each metacognitive factor. The assumption of homogeneity of variances was met for the pre-and post-test MALQ total scores (*p* = 0.59; *p* = 0.15). Table 4 shows that both the experimental and control groups improved metacognitive awareness by comparing the pre-test and post-test scores, with the largest improvements observed for Planning-evaluation and Problem-solving. Thereafter, an ANCOVA test was conducted to check the group effect among the five metacognitive factors, using post-test scores as the dependent variable and pre-test scores as the covariates (see Table 5). Table 5 demonstrates a significant group effect for the Planning-evaluation factor (*p* = 0.002), but no group effects were detected regarding the total scores or the remaining factors. As shown in Figure 2, the experimental group exhibited a greater improvement for Planning-evaluation than the control group after the treatment. The results indicated that the treatment effect only existed for the Planning-evaluation factor but did not exist for the other factors or the total metacognitive scores.

**Table 4:** Descriptive statistics of the metacognitive awareness factors.

**Source**	**Factors**	**Experimental group (*****n*** = **45)**		**Control group (*****n*** = **44)**	
		***M***	***SD***	***M***	***SD***
Pre-test	Planning- evaluation	2.73	0.75	2.59	0.81
	Directed attention	3.38	0.73	3.38	0.70
	Person knowledge	2.33	0.79	1.92	0.74
	Mental translation	2.86	0.79	2.89	0.82
	Problem- solving	2.84	0.74	2.58	0.83
	Total	2.83	0.40	2.67	0.38
Post-test	Planning- evaluation	3.40	0.57	3.05	0.59
	Directed attention	3.42	0.59	3.41	0.70
	Person knowledge	2.17	0.74	2.09	0.66
	Mental translation	2.61	0.72	2.83	0.68
	Problem- solving	3.28	0.55	3.06	0.61
	Total	2.98	0.38	2.89	0.31
Pre-post change	Planning- evaluation	0.67	0.73	0.46	0.87
	Directed attention	0.04	0.78	0.03	0.64
	Person knowledge	−0.16	0.58	0.17	0.91
	Mental translation	−0.25	0.90	−0.06	0.87
	Problem-solving	0.44	0.75	0.48	0.87
	Total	0.15	0.40	0.22	0.39

**Table 5:** ANCOVA results of the group effect on the metacognitive awareness factors.

**Source**	**Factors**	***df***	**Mean square**	***F***	***p***	**η^2^**
Group	Planning-evaluation	1	2.23	7.30	0.002^**^	0.08
	Directed attention	1	0.002	0.01	0.94	0.00
	Person knowledge	1	0.18	0.47	0.50	0.01
	Mental translation	1	1.06	2.39	0.13	0.03
	Problem-solving	1	0.59	1.97	0.16	0.02
	Total	1	0.02	0.18	0.68	0.00

** p < 0.01.

#### 4.2.2. LSQ results

**Figure 3:**
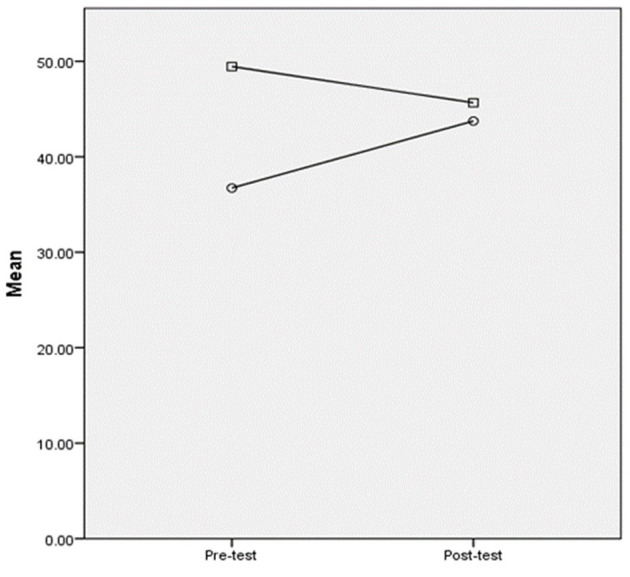
Plot comparing the pre-and post-test self-efficacy scores. ° = The experimental group; □ the control group.

Listening Self-efficacy Questionnaires (LSQ) were administered immediately after the pre-and post-listening tests. The description analysis is shown in Table 6. From Table 6 and Figure 3, it can be seen that the control group had higher self-efficacy scores as compared to the experimental group in the pre-test, while their scores were similar in the post-test. After training, the experimental group exhibited an increase in self-efficacy score, while the control group exhibited a decrease, which was similar to their performance in listening tests (see Figure 1). It can be implied that neither group was confident about their listening performance, given that their mean scores were <50 on a 100-scale measurement. ANCOVA was conducted to check the group differences by controlling for the pre-test scores as the covariate (see Table 7). Table 7 shows that there were no between-group differences in self-efficacy scores [*F*_(1,86)_ = 0.69, *p* = 0.41], suggesting no treatment effect on self-efficacy. We further conducted paired-sample *T*-tests to examine the within-group differences in self-efficacy scores. The experimental group's post-test scores were significantly higher than their pre-test scores [*t*_(1,44)_ = −2.72, *p* = 0.009], but no within-group differences were found in the control group [*t*_(1,43)_ = 1.61, *p* = 0.11]. This indicated that the experimental group developed listening self-efficacy after training, but superiority to the control group was not observed.

**Table 6:** Descriptive statistics of the self-efficacy scores.

**Tests**	**Experimental group (*****n*** = **45)**		**Control group (*****n*** = **44)**	
***M***	***SD***	***M***	***SD***
Pre-test	36.72	12.58	49.31	15.34
Post-test	43.74	15.00	45.63	13.95

**Table 7:** ANCOVA results of the group effect on the self-efficacy scores.

**Source**	***df***	**Mean square**	***F***	***p***	**η^2^**
Group	1	129.19	0.69	0.41	0.01
Pre-test	1	2,221.73	11.90	0.001^*^	0.12
Residual	86	186.73			

* p < 0.05.

In summary, the results of the listening tests and questionnaires showed that the experimental group receiving online metacognitive listening practice outperformed the control group on the listening tests. However, the results provided scant evidence for treatment effects on metacognitive awareness and self-efficacy. A significant treatment effect was only observed for the Planning-evaluation factor, but not for listening self-efficacy. The following analysis of the reflective notes and interviews from the experimental group can help enrich the above results.

### 4.3. Results from reflective notes and interviews

The analysis of the reflective notes and interview data provided us with more insights into the development of listening ability, metacognitive awareness, and self-efficacy.

First, learners acknowledged an improvement in listening comprehension ability and reported a metacognitive awareness of using strategies to solve problems in listening (e.g., planning, reflection, prediction, selective attention, directed attention, etc.), as seen in Table 8.

**Table 8:** Excerpts on listening comprehension and strategy use.

**Reflective notes**	**Codes**	**Themes**
After 2 weeks of listening practice, I became more interested in this kind of practice, and my listening ability has improved. (Sophia 2) Initially, I hardly followed the listening s*p*eed, but now I feel much better. (Alice 4)	Improvement in understanding fast speech	Improvement in listening comprehension
I can make predictions and recall some content and ideas before listening. (Leah 2)	Prediction strategy	Awareness of planning-evaluation
It is necessary to make a long-term listening plan and persist in it. (Harper 3)	Planning strategy	
I need to think about my listening problems and weakness carefully. (Camila 3)	Reflection strategy	
I should focus on the main idea and compare it with my predictions. (Ella 3)	Selective attention strategy	
**Interviews**	**Codes**	**Themes**
(Now) While listening, I can grasp more details and become more patient than before. (Ella)	Improved comprehension ability	Improvement in listening comprehension
Through online listening practice, I realize that listening does not just mean listening itself, but involves many activities like prediction and reflection. (Mila)	Prediction and reflection strategy	Awareness of planning-evaluation
Reflection must be helpful. Through reflection, I can find some potential problems I did not realize before. (Nova)	Reflection strategy

The names reported here are pseudonyms. The numbers after the names denote the time of the note within seven notes in 14 weeks.

In the reflective notes and interviews, the learners (e.g., Sophia, Alice, and Ella) reported progress in their listening comprehension ability. Moreover, some learners (e.g., Leah, Harper, Camila, Ella, Mila, and Nova) indicated the development of listening strategies, especially those about planning (e.g., planning, prediction, and selective attention) and reflection (evaluation). Also, Mila revealed that she was developing an awareness of prediction (planning) and reflection (evaluation) during the online listening practice. These accounts illustrate the development of listening comprehension and Planning-evaluation derived from listening tests and the MALQ questionnaires.

Second, as seen in Table 9, learners perceived uncertainty in listening confidence and problems in anxiety. Meanwhile, there is a fluctuating awareness of using mental translation among these learners.

**Table 9:** Excerpts on confidence, anxiety, and mental translation.

**Reflective notes**	**Codes**	**Themes**
Over a long period of listening practice, I gradually obtained more confidence. (Leah 4)	Improved listening confidence	Uncertainty in confidence and problems with anxiety
I have trouble listening to some new words, which causes me to become anxious, distracted, and less confident. (Audrey 3)	Existing problems with anxiety and confidence	
With more practice and awareness, I did not translate mentally as much. (Sophia 4)	Improvement in avoiding mental Translation	Fluctuation in mental translation
I don't think metal translation is bad. The amount of time required for translation will decrease as we become more proficient. The translation itself is a just kind of understanding. (Ruby 2)	Benefits of mental translation	
I do not mentally translate short and simple sentences, but I do it for long and complex sentences. (Delilah 5)	Using mental translation in different contexts	
**Interviews**	**Codes**	**Themes**
I feel a little improvement in confidence. However, I still fail to do the tasks well in the tests. (Harper)	Slight improvement in confidence	Uncertainty in confidence and problems with anxiety
Once I fail to understand some parts of listening, I feel very anxious. (Sophia)	Existing problems with anxiety	
I feel it is impossible to avoid mental translation. My first task in listening is to translate what comes into my head. (Noah)	Impossibility of avoiding mental translation	Fluctuation in mental translation

The names reported here are pseudonyms. The numbers after the names denote the time of the note within seven notes in 14 weeks.

According to Audrey, Harper, and Sophia's statements, their listening confidence and anxiety were affected by the specific listening activities. For them, difficult listening tasks or tests seemed to decrease their listening confidence and increase their listening anxiety. Ruby appeared to support the use of mental translation after weighing its value, whereas Delilah held a neutral attitude toward it. Noah admitted the impossibility of avoiding mental translation. Learners appeared to have differing views about the value of avoiding mental translation, the benefits of which were not clearly shown.

In summary, the qualitative results suggested that the experimental group confirmed their progress in improving their listening ability and their metacognitive awareness of adopting strategies to manage the listening process and tackle listening problems. However, participants were uncertain regarding listening confidence, anxiety, and mental translation, which may explain the limited effects of the treatment on metacognition and self-efficacy, as reported in the quantitative results.

## 5. Discussion

### 5.1. Listening comprehension ability

Responding to the first research question, the experimental group under the online metacognitive listening practice significantly outperformed the traditional group on the final listening test, suggesting a positive treatment effect. This result supports the previous conclusion (e.g., Vandergrift and Tafaghodtari, 2010; Cross, 2011; Bozorgian, 2014; Bozorgian and Alamdari, 2017) that increasing metacognitive awareness during the listening process does have value in improving L2 listening comprehension ability. Vandergrift and Tafaghodtari (2010) found that L2 listeners receiving metacognitive instruction significantly outperformed those of traditional listening instruction in L2 listening. They indicated that the frequent involvement in metacognitive processes enables learners to acquire the implicit knowledge of listening strategies and metacognition progressively. With this tacit knowledge, learners can regulate listening processes and establish learning automatization (Wenden, 1998), thus acting as expert listeners (Field, 2008). Similarly, in the present study, learners were allowed to constantly engage in the cycle of metacognitive processes via online listening practice, which helped them form implicit listening knowledge and facilitated learner autonomy and listening development.

This study also alludes to the critical role of self-reflection tasks, although some earlier studies (e.g., Bozorgian and Alamdari, 2017; Mahdavi and Miri, 2019) highlighted the role of discussion in metacognitive instruction. While learners have little opportunity for discussion in a self-directed online setting, they can gain metacognition awareness from reflections and external feedback. Graham and Macaro (2008) indicated that reflective diaries with written feedback could assist listeners in establishing a link between strategy use and listening performance. Goh and Vandergrift (2021) suggested that guided reflection tasks, either from discussion or self-reflection, are helpful in eliciting listeners' metacognitive knowledge. With increased metacognitive knowledge, learners can monitor their listening process and attain listening improvements independently.

### 5.2. Listening metacognitive awareness

For the second research question, it was found that the experimental group significantly outperformed the traditional group in only one factor of metacognitive awareness, namely, Planning-evaluation. The significant improvement in Planning-evaluation is consistent with the study of Bozorgian (2014), which showed that metacognitive instruction can significantly improve Iranian EFL learners' two sub-factors of metacognitive awareness, i.e., Planning-evaluation and Problem-solving. Similarly, limited improvement in metacognitive awareness was also noted in Vandergrift and Tafaghodtari (2010), which found that the positive effect of metacognitive instruction is only associated with Problem-solving. Given the limited between-group differences, they inferred that the control group's improvement in metacognitive awareness might be attributed to exposure to the metacognitive listening questionnaire. This explanation can also be applied in this study, as the control group improved certain metacognitive awareness factors (as shown in Table 4).

The limited development of metacognitive awareness may also be related to the task settings of the Metacognitive Pedagogical Cycle (MPC) (Vandergrift, 2004, 2007). Most MPC tasks focus on the training of planning, verification, and evaluation strategies, thus highlighting the Planning-evaluation factor more than other factors, such as Directed Attention and Mental Translation. That is, listeners have more opportunities to practice the strategy of planning and evaluation under MPC. The experimental group's success in Planning-evaluation, not in the other factors, may be explained by such task settings. Furthermore, explanations can be framed in terms of different weights in the sub-factors of metacognitive awareness. Azmee (2022) discovered that certain factors, such as Planning-evaluation, Directed Attention, and Problem-Solving, strongly link with listening ability. In contrast, others, such as Person Knowledge and Mental Translation, only have marginal relationships with L2 listening ability.

In addition, the reflective notes and interviews from the experimental group may help explain the partial development of metacognitive awareness. These learners reported an awareness of certain strategies, such as planning and reflection but also had an uncertain attitude toward the effectiveness of mental translation. Nevertheless, it made sense that some learners retained the use of mental translation given that the use of L1 is often regarded as a helpful strategy for L2 reading and writing. For instance, Kern (1994) indicated that the use of L1 can assist learners in overcoming cognitive limits and removing affective barriers when reading, e.g., L2 writers often fall back on L1 in translating keywords (Sasaki, 2000) and thinking about the writing process (Cumming, 1990). Even if translation mentally can impede comprehension fluency, it may be helpful to jot down keywords and it can help alleviate anxiety (as shown in some learners' notes), as seen in L2 reading and writing.

### 5.3. Listening self-efficacy

As regards the third research question, the online metacognitive listening practice did not yield a significant treatment effect on listening self-efficacy, although within-group differences were observed in the experimental group. Nevertheless, this result agrees with the findings of Taguchi (2017) and Milliner and Dimoski (2021). Both investigated the effects of metacognitive instruction on listening self-efficacy with Japanese EFL learners. In Taguchi's study, both the experimental and control groups made improvements in listening self-efficacy, although no treatment effect was found. The author suggested that the improvement in self-efficacy in both groups was due to increased listening practice, which contributed to an increase in successful listening (mastery) experiences (Bandura, 1994) and listening self-efficacy. Milliner and Dimoski's (2021) findings are similar to those of the present study, i.e., no treatment effect for self-efficacy was detected, but the experimental group showed significant improvement in listening self-efficacy. The authors further indicated that the partial improvement in self-efficacy may help sustain the development of listening comprehension over time, given that self-efficacy beliefs can increase learners' efforts and perseverance. Since the present study and Milliner and Dimoski's (2021) study used the same questionnaire from Graham and Macaro (2008), the similar results from the two studies increase the replicability and further confirm the limited effects of the metacognitive intervention on self-efficacy.

Furthermore, listening self-efficacy appears to be task dependent. Some learners indicated that their listening confidence and anxiety are influenced by the difficult listening tasks with quick speech rates and unfamiliar words, or high-stake tests. It is plausible that difficult listening tasks reduce opportunities for successful listening experiences, i.e., mastery experiences (Bandura, 1994), thus weakening their self-efficacy beliefs. Therefore, the control group with limited intervention in improving metacognitive awareness in terms of self-managing and evaluating the listening process may have been blocked by difficult listening tasks, leading to a decline in self-efficacy. However, due to the higher pre-test scores of the control group in the present study, we cannot rule out the influence of the ceiling effect on the post-test scores. Therefore, further studies aimed at examining changes in self-efficacy should consider investigating learner groups with a similar level of self-efficacy before the study.

## 6. Conclusion

This study adds to the limited research on online metacognitive listening intervention and investigates its effect on listening comprehension, metacognitive awareness, and self-efficacy. The results demonstrate that the self-directed online listening practice deployed under the Metacognitive Pedagogical Cycle (MPC) can significantly improve Chinese intermediate EFL learners' listening comprehension. This finding suggests that the MPC is a promising approach for improving L2 listening in self-directed learning contexts. However, the treatment effect on metacognitive awareness and self-efficacy is not conclusive. Learners significantly improved in the only sub-factor of metacognitive awareness, i.e., Planning-evaluation, but not in the listening self-efficacy.

This study also makes some noteworthy methodological contributions. Firstly, we adapted the metacognitive pedagogical cycle into sets of self-directed online listening practice exercises, so that learners can experience metacognitive listening processes on their own. Second, we attempted to balance the efforts of the experimental and control groups by giving more practice to the control group, due to the lower task complexity. Third, the study used various data sources (i.e., listening tests, questionnaires, reflective notes, and interviews) to triangulate the findings, increasing the study's trustworthiness.

One limitation of the study is that we conducted interviews only with the experimental group, which may have resulted in missing information about the perceived change in the control group. To gain a more complete understanding of the differences between the two groups, future research should include interviews from both groups. Besides, the sample size of 89 participants in this study is relatively modest, with <50 in the experimental group, and future investigations may consider expanding the sample size to enhance the reliability. Additionally, the study removed the discussion part from the MPC due to the difficulty of implementation in an online setting, which was partly redeemed with extra reflection activities. Since previous studies (e.g., Bozorgian and Alamdari, 2017; Mahdavi and Miri, 2019) have highlighted the crucial role of discussion in developing listeners' metacognitive awareness, further studies could explore the possibility of including synchronous or asynchronous discussions during online listening.

## Data availability statement

The raw data supporting the conclusions of this article will be made available by the authors, without undue reservation.

## Ethics statement

The studies involving humans were approved by School of Foreign Languages, Shaoguan University. The studies were conducted in accordance with the local legislation and institutional requirements. The participants provided their written informed consent to participate in this study.

## Author contributions

TP: Conceptualization, Data curation, Formal analysis, Funding acquisition, Investigation, Writing—original draft, Writing—review & editing. JS: Conceptualization, Methodology, Supervision, Writing—review & editing. HL: Writing—review & editing.
